# The Shp2-induced epithelial disorganization defect is reversed by HDAC6 inhibition independent of Cdc42

**DOI:** 10.1038/ncomms10420

**Published:** 2016-01-19

**Authors:** Sui-Chih Tien, Hsiao-Hui Lee, Ya-Chi Yang, Miao-Hsia Lin, Yu-Ju Chen, Zee-Fen Chang

**Affiliations:** 1 Institute of Biochemistry and Molecular Biology, National Yang-Ming University, No. 155, Section 2, Linong Street,Taipei 11221, Taiwan; 2 Department of Life Sciences and Institute of Genome Sciences, National Yang-Ming University, No. 155, Section 2, Linong Street,Taipei 11221, Taiwan; 3 Institute of Chemistry, Academia Sinica, 128 Academia Road, Section 2, Nankang, Taipei 11529, Taiwan

## Abstract

Regulation of Shp2, a tyrosine phosphatase, critically influences the development of various diseases. Its role in epithelial lumenogenesis is not clear. Here we show that oncogenic Shp2 dephosphorylates Tuba to decrease Cdc42 activation, leading to the abnormal multi-lumen formation of epithelial cells. HDAC6 suppression reverses oncogenic Shp2-induced multiple apical domains and spindle mis-orientation during division in cysts to acquire normal lumenogenesis. Intriguingly, Cdc42 activity is not restored in this rescued process. We present evidence that simultaneous reduction in myosin II and ERK1/2 activity by HDAC6 inhibition is responsible for the reversion. In HER2-positive breast cancer cells, Shp2 also mediates Cdc42 repression, and HDAC6 inhibition or co-suppression of ERK/myosin II promotes normal epithelial lumen phenotype without increasing Cdc42 activity. Our data suggest a mechanism of epithelial disorganization by Shp2 deregulation, and reveal the cellular context where HDAC6 suppression is capable of establishing normal epithelial lumenogenesis independent of Cdc42.

## Abstract (web summary)

Cdc42 activity is important for apical-basal epithelial polarity. Here, the authors show that Shp2 disrupts Cdc42 activation, and by reducing the expression of histone deactylase 6, restores epithelial lumen formation in a cdc42-independent manner.

## Graphical abstract

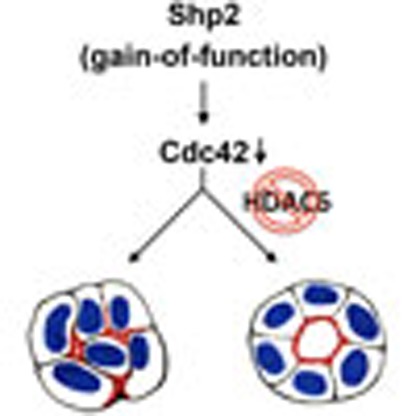

Shp2 is a ubiquitously expressed non-receptor protein tyrosine phosphatase^1^, and hyper-activation of Shp2 is related to pathogenesis of many diseases including developmental disorders, cancers and metabolic diseases^2^,^3^,^4^. Shp2 contains two Src homology-2 (N-SH2 and C-SH2) domains, and its catalytic domain is in a closed conformation due to the intramolecular interaction with the N-SH2 domain^5^, restricting its phosphatase function. Germline or somatic gain-of-function mutation such as E76G in N-SH2 domain that disrupts this closed conformation has been shown to play a causal role in Noonan syndrome, leukaemia and solid tumours^6^,^7^,^8^. In addition, a variety of signalling via disease-associated tyrosine kinases also deregulate Shp2. For example, HER2 or BCR-ABL causes constitutive tyrosine phosphorylation of Gab2 that interacts with Shp2, thus disrupting its auto-inhibition to enhance its phosphatase function^9^,^10^,^11^.

It has been shown that *Shp2* homozygous deletion results in embryonic lethality due to the lack of ERK activation^12^, whereas a number of Noonan syndrome mouse models have demonstrated the contribution of Shp2-mediated ERK hyper-activation to the disease phenotype^13^,^14^,^15^. These mice studies highlight the essential regulation of ERK by Shp2. In cancer, gain-of-function Shp2 also causes hyper-activation of Ras/Raf/ERK signalling, leading to the loss of growth control^10^,^16^. In addition to growth aberration, Shp2-mediated oncogenesis has been shown to involve the loss of epithelial organization^17^. In severe gastric diseases associated with *Helicobacter pylori* infection, it has been shown that CagA, a pathogenic factor, binds and activates Shp2 to impair apical–basolateral polarity and cell–cell junctions, generating hummingbird phenotype^18^,^19^. In breast cancer study, knockdown of Shp2 in MCF10A-HER2/3 cells or BT474 cells is sufficient to rescue hollow lumen phenotypes in three-dimensional (3D) culture^20^,^21^,^22^. However, it remains elusive how oncogenic Shp2 acts to cause epithelial disorganization. Disruption of normal epithelial morphogenesis is known to cause various diseases and tumorigenesis^23^,^24^,^25^. Understanding the underlying mechanism might suggest new therapeutic target for the treatment of diseases involving Shp2 deregulation.

Normal epithelial lumenogenesis requires the apical–basal polarity to position apical initiation and the spindle orientation in divisions for developing normal lumen^26^. In establishing apical–basal polarity, Cdc42, a member of Rho GTPase family, is activated during apical vesicle trafficking to mediate apical activation of aPKC via complex formation with Par3/Par6 for a proper apical domain formation^27^,^28^,^29^. In this report, we compare Cdc42 activity and lumen formation in Madin–Darby Canine Kidney (MDCK) cells expressing similar level of wild-type (WT) Shp2 and oncogenic Shp2-E76G (ref. 7) in 3D culture. Our data demonstrated that Shp2-E76G expression reduced Cdc42 activity, leading to multi-lumen cyst formation as seen in MDCK with Cdc42 inactivation^30^. The mechanistic investigation indicates that the function of Tuba, a guanine exchange factor of Cdc42 (refs 31, 32), is highly dependent on site-specific tyrosine phosphorylation. Expression of oncogenic Shp2 suppressed phosphorylation of Tuba to decrease Cdc42 activity, therefore, leading to defect in lumen formation. Moreover, the repression of Cdc42 is also found in HER2-positive breast cancer cells.

HDAC6 is a deacetylase of tubulin and its inhibition leads to microtubule hyper-acetylation^33^. In this study, we found that HDAC6 suppression restores normal epithelial lumen formation in MDCK cells expressing oncogenic Shp2 cells. Intriguingly, this reversion is independent of Cdc42 signal or aPKC activity. It has been reported that depletion of HDAC6 attenuates epidermal growth factor-induced ERK activation^34^. We have previously shown that HDAC6 inhibition reduces ERK1/2 phosphorylation while expression of tubulin mutant deficient of acetylation has an inverse effect, indicating the repression of ERK activation by microtubule acetylation^35^. Meanwhile, a recent report has demonstrated that microtubule acetylation by HDAC6 inhibition decreases myosin II activity by promoting myosin phosphatase binding to myosin light chain (MLC)^36^ In agreement, we found that HDAC6 inhibition causes a concurrent reduction in ERK and myosin II activity. Our data demonstrate that their co-suppression prevents lumen aberration not only in MDCK but also HER2-positive breast cancer BT474 cells without increasing Cdc42 activity. Thus, the suppression of HDAC6 allows the apical–basal polarity formation in a context deficient of Cdc42-mediated signal through attenuating ERK and myosin II.

## Results

### Oncogenic Shp2 suppresses Cdc42 and lumenogenesis in MDCK

**Figure 1:**
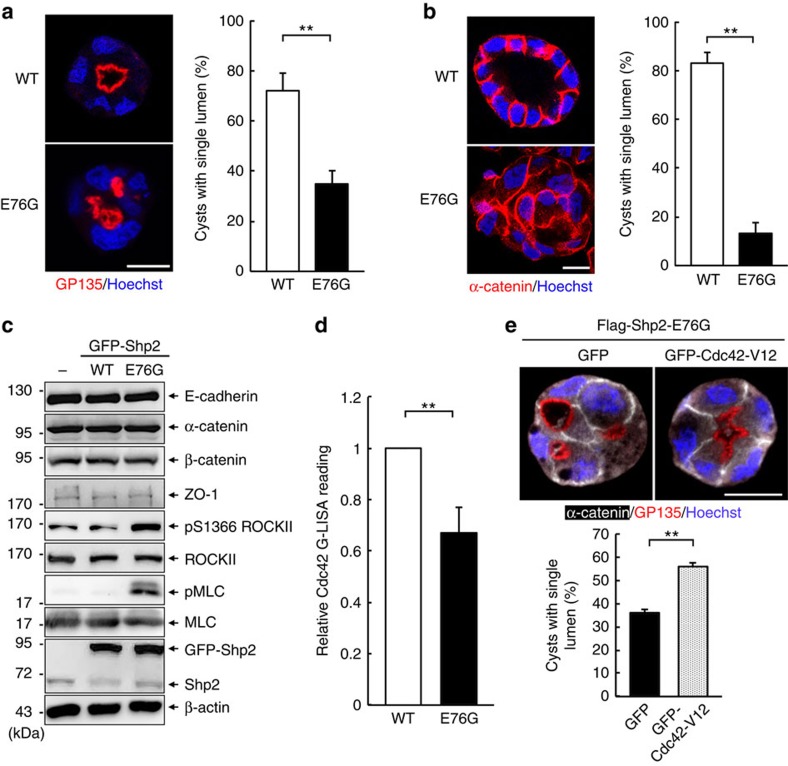
Expression of oncogenic Shp2 impairs normal epithelial lumen formation through reducing Cdc42 activity. MDCK cells expressing GFP-Shp2-WT and GFP-Shp2-E76G were embedded in Matrigel. (**a**) After 2 days, cysts were extracted and fixed for immunofluorescence (IF) staining with antibodies as indicated. The percentage of cysts with single lumen is shown on the right (312–486 cysts were counted in each experiment, *n*=3). (**b**) After 7 days, cysts were extracted for IF staining, scale bar, 10 μm. Right panel shows the percentages of cysts with one single interior hollow lumen (584–767 cysts were counted in each experiment, *n*=3). (**c**) The 7-day cysts were harvested for western blot analysis with the indicated antibodies. (**d**) Cysts (2 days) were extracted for measuring the endogenous Cdc42 activity by G-LISA assay. (**e**) MDCK cells expressing Flag-Shp2-E76G were transfected with the expression vector of GFP or GFP-Cdc42-V12 and then embedded in Matrigel for 2 days. The formed cysts were fixed for IF stained. The percentage of cysts with single apical patch is shown below (203–241 green-positive cysts were counted in each experiment, *n*=3). Scale bars, 10 μm. All values are mean±s.d. from three independent experiments; statistical significance was analysed by two-tailed unpaired Student's *t*-test. *P<0.05, **P<0.01.

We have previously established MDCK stable clones that express similar level of GFP-Shp2-WT and GFP-Shp2-E76G mutant^35^. To know the effect of oncogenic Shp2-E76G in epithelial organization, we embedded these cells in Matrigel for 3 days. Immunofluorescence staining in Shp2-WT cysts showed that GP135, the apical marker, fused with the apical plasma membrane to form a single apical domain (Fig. 1a). In contrast, Shp2-E76G-expressing cysts displayed random distribution of GP135 vesicles, an indication of early abnormality in apical domain formation (Fig. 1a). Prolonged incubation of cysts in Matrigel for 7 days, WT cells formed cysts with a single hollow lumen in spherical structure, while E76G cysts were in irregular shape without central lumen formation (Fig. 1b). Western blot analysis of these cysts showed the protein levels of adherens junction components unaffected by the expression of oncogenic Shp2-E76G (Fig. 1c). It was noted that ROCKII was more active in these cysts as revealed by an increase in autophosphorylation of ROCKII at S1366 (ref. 37). In agreement, phosphorylation of MLC was also elevated in these cysts. This observation is consistent with our previous finding that Shp2 upregulates ROCK activation^38^. However, treatment of E76G cells with Y27632, a specific ROCK inhibitor^39^, was unable to restore normal lumen formation (Supplementary Fig. 1).

Since Cdc42 inactivation also caused multi-lumen phenotype in MDCK cells^30^, we then analysed the activity of Cdc42 in these cysts. Data showed that E76G cysts had lower activity of Cdc42 than WT cysts (Fig. 1d). Expression of GFP-Cdc42-V12, a dominant active mutant, partially restored the single lumen formation in Shp2-E76G cells (Fig. 1e). These results suggest that lumen defect in Shp2-E76G cysts involves the suppression of Cdc42 activity.

### Shp2 dephosphorylates Tuba to cause a lumen defect

**Figure 2:**
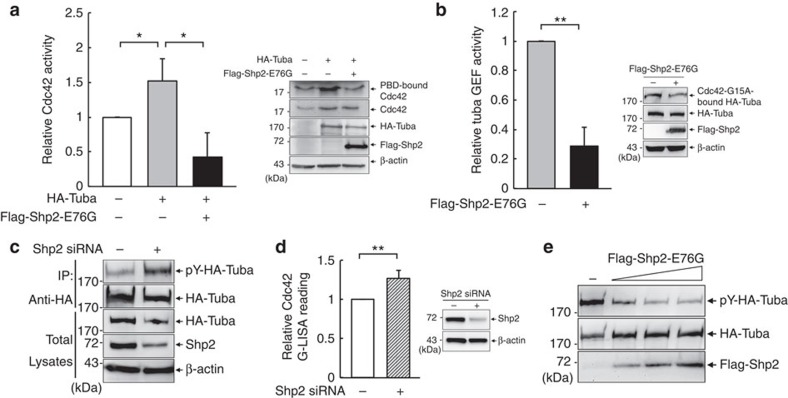
Regulation of Tuba-mediated Cdc42 activation by Shp2. (**a**,**b**) HEK293T cells were transfected with HA-Tuba in the presence or absence of Flag-Shp2-E76G as indicated. Cells were harvested for (**a**) GST-PBD pull-down assay to determine endogenous Cdc42 activity, and (**b**) GEF activity of HA-tuba by GST-Cdc42-G15A pull-down assay. The representative Western blots for the assays are shown on the top. The relative Cdc42 and HA-Tuba GEF activity from three independent experiments are shown below. Data are presented as mean±s.d.; Two-tailed unpaired Student's *t*-test was performed, *P<0.05, **P<0.01. (**c**) HEK293T cells were transfected with or without Shp2 siRNA for 3 days, followed by transfection with the expression construct of HA-Tuba. HA-Tuba protein was immunoprecipitated with anti-HA beads for western blotting with anti-phosphotyrosine (4G10) and anti-HA antibodies. The total lysates were also subjected to Western blot analysis as indicated. (**d**) Cdc42 activity was measured by G-LISA assay in cells with and without Shp2 knockdown. Values are mean ±S.D from three independent experiments. **P<0.01 based on 2-tailed unpaired Student's *t* test. (**e**) Immunoprecipitated HA-Tuba protein from pervanadate-treated cells was incubated with increasing amounts of immunopurified Flag-Shp2-E76G protein at 30 °C for 30 min and analyzed by western blotting as indicated.

To understand the molecular mechanism for Shp2-E76G-induced downregulation of Cdc42 activity, we tested the effect of Shp2-E76G expression on the function of Tuba, an essential guanine exchange factor (GEF) in Cdc42 activation during lumen formation^32^. Due to the low transfection efficiency of MDCK cells, we used HEK293T cells to analyse the regulation of endogenous Cdc42 activity by Tuba in relation to oncogenic Shp2. Either overexpression of Tuba or Vav1, another Cdc42 GEF, was able to increase Cdc42 activity, but co-expression of Shp2-E76G abolished only Tuba- but not Vav1-induced Cdc42 activation (Fig. 2a and Supplementary Fig. 2). This implies that Shp2 specifically represses Tuba-mediated Cdc42 activation. The assessment of GEF activity was further performed by GST-Cdc42-G15A pull-down analysis^40^. The results showed that GEF activity of hemagglutinin(HA)-Tuba was significantly reduced by expression of Shp2-E76G (Fig. 2b). Moreover, Shp2 knockdown increased tyrosine phosphorylation level of Tuba and Cdc42 activity (Fig. 2c,d). We then tested whether Tuba is a direct substrate of Shp2. Incubation of phospho-Tuba isolated from cell lysates with Shp2 immunocomplex abolished the intensity of tyrosine phosphorylation of Tuba (Fig. 2e), suggesting the substrate–phosphatase relationship between phospho-Tuba and Shp2.

**Figure 3:**
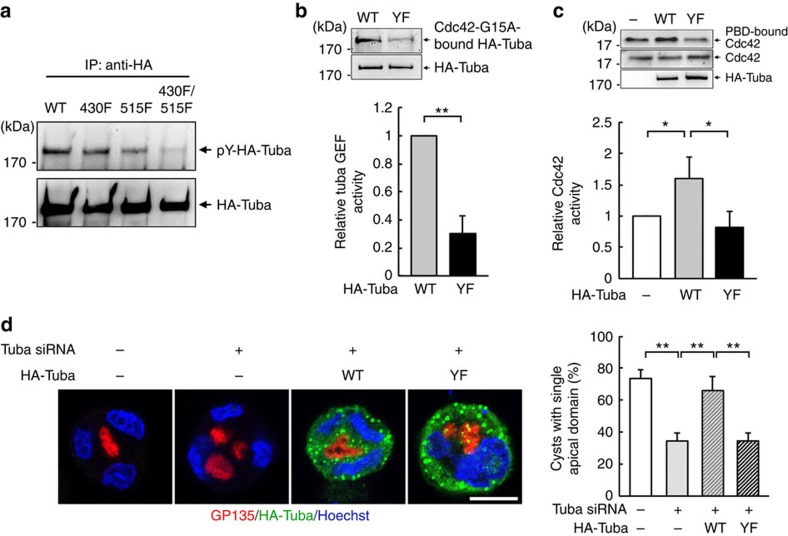
Dephosphorylation at Y430/Y515 by oncogenic Shp2 impairs Tuba-mediated Cdc42 activation and normal lumen formation. (**a**) HEK293T cells were transiently transfected with HA-Tuba of WT and various mutants carrying Y→F mutation as indicated. Cells were treated with pervandate (50 μM) for 5 min before harvested for immunoprecipitation with anti-HA antibody. The immunoprecipitates were analysed by western blot with anti-phosphotyrosine (4G10) antibody followed by reporbing with anti-HA antibody. (**b**,**c**) HEK293T cells were transfected with control vector or HA-Tuba WT or YF (Y430/515F) mutant and harvested for (**b**) measuring GEF activity of HA-tuba by GST-Cdc42-G15A pull-down assay, and (**c**) endogenous Cdc42 activity by GST-PBD pull-down assay. The relative Cdc42 and HA-Tuba GEF activity from three independent experiments are shown. (**d**) MDCK cells were co-transfected with Tuba siRNA and WT or 430/515YF mutant of HA-Tuba and then cultured in Matrigel for 2 days. Cysts were extracted for IF staining with anti-GP135 (red) and anti-HA (green) antibodies and Hoechst (blue), scale bar,10 μm. The percentage of cysts with single apical patch is shown on the right (113–179 cysts were counted in each experiment, *n*=3). Data are presented as mean±s.d. from three independent experiments; two-tailed unpaired Student's *t*-test. *P<0.05, **P<0.01.

A number of phosphatome studies have detected multiple phosphorylation sites in Tuba protein^41^,^42^,^43^. According to these identified phosphorylated sites, we generated phenylalanine mutants at Y430, Y456, Y515 and Y1433 sites. Introduction of Y456F or Y1433F mutation did not affect the phosphorylation level (Supplementary Fig. 3a). As a contrast, Y430F and Y515F mutation reduced phosphorylation level of Tuba protein (Fig. 3a). Tuba mutant with double mutation at Y430 and Y515 residues was further generated. The phosphorylation level of this Y430F/Y515F Tuba mutant was significantly decreased. Cdc42-G15A pull-down analysis showed that the GEF activity of this HA-Tuba YF mutant was very low (Fig. 3b). In agreement, Cdc42 activity in cells expressing Y430F/Y515F Tuba was lower than that in WT Tuba cells (Fig. 3c), indicating that the phosphorylation of these two tyrosine residues positively regulates the function of Tuba in Cdc42 activation. In addition to Y430/Y515, our mass spectrometric analysis of phosphopeptides of Tuba showed phosphorylation of Y1157 sensitive to Shp2-E76G-mediated dephosphorylation (Supplementary Fig. 3B). We further decreased the expression level Tuba in MDCK cells by small interfering RNA (siRNA) transfection and re-introduced HA-tagged WT and YF siRNA-resistant mutant in these cells to evaluate the importance of phosphorylation at these sites in apical domain formation. The analysis of cysts showed that Tuba knockdown caused multiple apical domain. Re-introduction of HA-Tuba expression vectors that are resistant to siRNA of Tuba indicated that WT, but not double YF mutant of Tuba, restored single apical domain in 3D culture (Fig. 3d). In conclusion, deregulation of Shp2 causes hypophosphorylation of Tuba, in turn decreasing its GEF function in Cdc42 activation to impair normal epithelial lumen formation.

### HDAC6 suppression reverses epithelial disorganization

**Figure 4:**
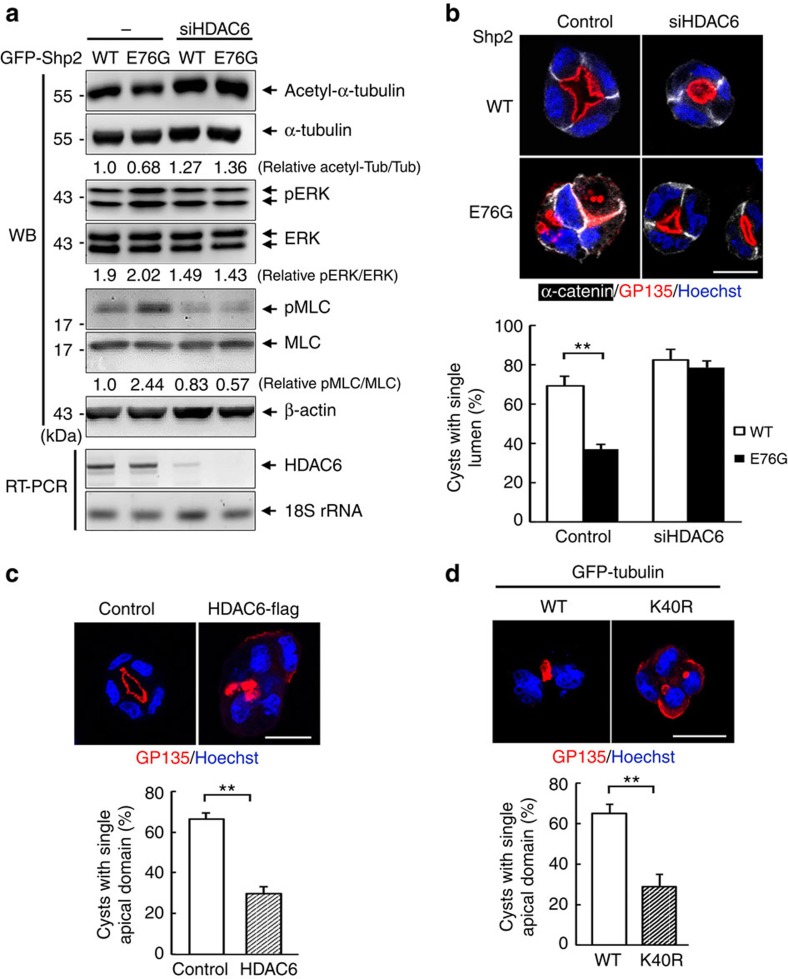
Role of HDAC6 in lumen formation and Microtubule acetylation in normal lumenogenesis. MDCK cells expressing GFP-Shp2-WT and GFP-Shp2-E76G were transfected with HDAC6 siRNA. (**a**) Western blot analysis. RT-PCR shows the knockdown effect.(**b**) Cells grown in Matrigel for 2 days were fixed for immunostained with antibodies as indicated. The percentage of cysts with single apical domain is shown in the right (516–641 cysts were counted in each experiment, *n*=3). (**c**) Control MDCK cells were co-transfected with control vector or HDAC6-Flag with GFP-C1 (amount of DNA ratio at 3:1). These cells were embedded into Matrigel for 2 days. Cysts were fixed for IF staining as indicated. The percentage of GFP positive cysts with single apical domain is shown at the bottom (167–193 green cysts were counted in each experiment, *n*=3). (**d**) Control MDCK cells were transfected with GFP-Tubulin WT or K40R mutant and embedded in Matrigel for 2 days. Cysts were fixed for IF staining with anti-GP135 (red) and Hoechst (blue), scale bar, 10 μm. The percentage of cysts with single apical domain is shown below (104–121 green-positive cysts were counted in each experiment, *n*=3). Values are mean±s.d. from three independent experiments. **P<0.01 based on two-tailed unpaired Student's *t*-test.

Specific membrane vesicle trafficking that moves along microtubule controls epithelial basal–apical polarity^27^. Microtubule acetylation regulated by HDAC6 influences microtubule stability^33^,^35^. To learn the relationship between microtubule acetylation and lumen defect in 3D culture, we decreased the expression level of HDAC6 by siRNA transfection in Shp2-E76G cells. Notably, this treatment increased microtubule acetylation with concurrent reduction in phosphorylation of both ERK1/2 and MLC (Fig. 4a). By examining the 3-day cysts, we found that HDAC6 knockdown restored the apical delivery of GP135 in Shp2-E76G cells to have a single lumen (Fig. 4b). Conversely, overexpression of HDAC6 in control MDCK was sufficient to cause multiple apical domain formation (Fig. 4c). To assure the specific involvement of microtubule acetylation in determining lumen morphogenesis, we further expressed unacetylable tubulin K40R mutant in control MDCK cells to specifically decrease microtubule acetylation. The K40R-tubulin mutant cyst also displayed multi-lumen phenotype, indicating a critical role of microtubule acetylation in normal lumenogenesis (Fig. 4d). It is worth of noting that overexpression of HDAC6 elevated phosphorylation of ERK1/2 and MLC (Supplementary Fig. 4). In conclusion, suppression of HDAC6 rescues normal lumen formation in Shp2-E76G cells, accompanied by microtubule hyperacetylation and co-suppression of ERK and myosin II activity. Conversely, HDAC6 overexpression that causes microtubule hypoacetylation is sufficient to block this process in the control MDCK cells.

### Blocking HDAC6 causes Cdc42 dispensable for lumenogenesis

**Figure 5:**
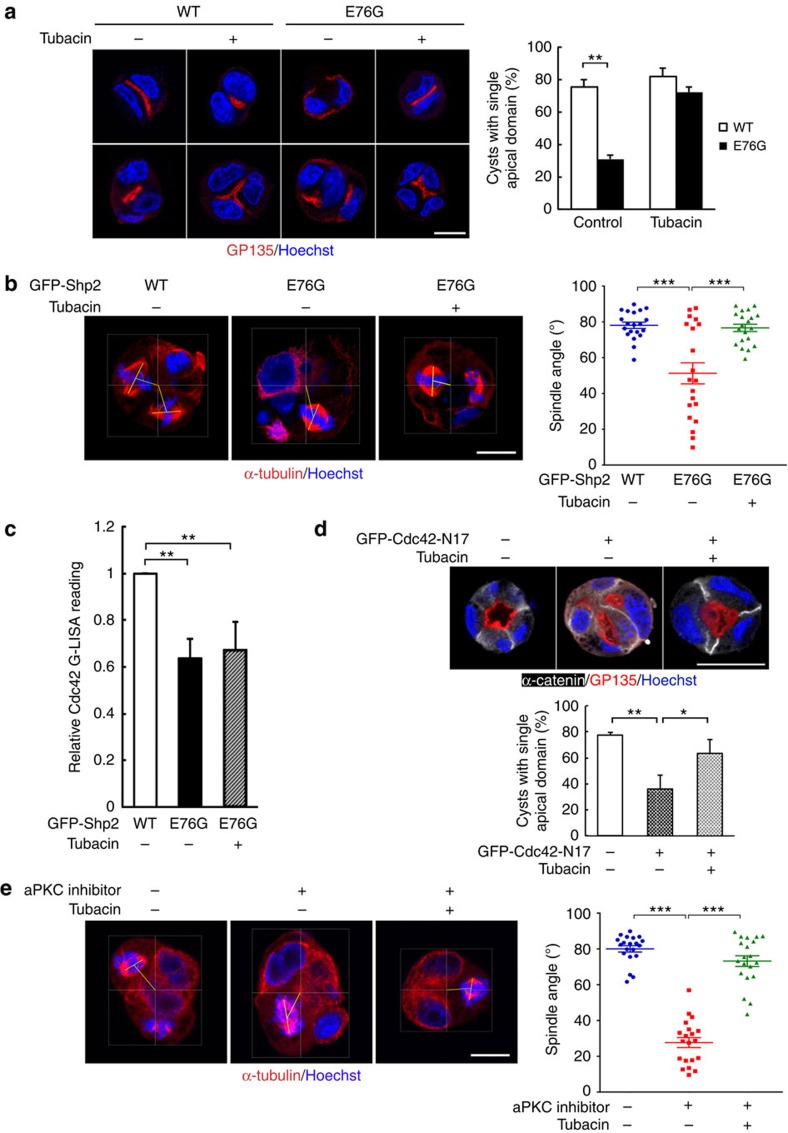
HDAC6 inhibition allows normal apical domain formation independent of Cdc42 signal. (**a**,**b**) MDCK expressing GFP-Shp2-WT or GFP-Shp2-E76G were embedded in Matrigel and treated with or without 1 μM of tubacin for 24 h. (**a**) Cyst were extracted for IF staining analysis, and the percentage of cysts with single apical domain is shown in the right (514–637 cysts were counted in each experiment, *n*=3.) (**b**) Cyst cultures were further treated with 10 μM of RO-3306 for 16 h to synchronize cells at the G2/M phase, and then released by replacement with normal medium for 40–50 min. Cysts were extracted for spindle analysis by α-tubulin IF and Hoechst staining. The angle between the spindle axis (thick line) and the thin line connecting the centre of cyst and the midpoint of spindle axis was measured. Scatter diagram of metaphase spindle angles are shown on the right. The data are presented as mean±s.e.m.; (*n*=20). (**c**) MDCK cells stably expressing GFP-Shp2-WT or E76G mutant were treated with tubacin (1 μM) for 2 days and harvested for Cdc42 activity by G-LISA assay. (**d**) GFP or GFP-Cdc42-N17-expressing MDCK cells in Matrigel and treated with tubacin (1 μM) for 2 days. Cysts were fixed and IF stained with antibodies as indicated. The percentage of cysts with single lumen is shown at the bottom (100 green-positive cysts were counted in each experiment, *n*=3). Values are mean±s.d. from three independent experiments.*P<0.05, **P<0.01 based on two-tailed unpaired Student's *t*-test. (**e**) MDCK cells in Matrigel were treated with 20 μM of aPKC inhibitor with or without tubacin (1 μM) for 24 h, followed by synchronization with RO-3306 for spindle observation. Right panel shows the spindle angle and data were presented as (**b**). ***P<0.001 based on two-tailed Student's *t*-test. Scale bars, 10 μm.

It is known that the first cell division of MDCK cells in Matrigel defines the location of the apical plasma membrane domain^30^. Shp2-E76G cells at two-cell stage showed multiple apical vacuoles formation, as indicated by GP135 staining. Treatment of these cells with tubacin, a highly potent and cell-permeable inhibitor of HDAC6 (ref. 44), gave a single apical domain in two-cell and four-cell stage (Fig. 5a), leading to normal lumen formation in 7 days of 3D culture (Supplementary Fig. 5).

During the metaphase of a second or later division round, proper spindle orientation in cyst is critical for normal lumenogenesis dependent on Cdc42/Par6/Par3/aPKC complex^32^,^45^,^46^. We then measured the angle between the spindle axis and the line connecting the centroid of cyst and midpoint of spindle axis. In Shp2-WT cysts, the line is predominantly perpendicular to the spindle axis. Oncogenic Shp2-E76G expression caused metaphase spindles tilted in a wide range. The average angle between the spindle axis and the line was about 50°, while in tubacin-treated cells, the alignment of the metaphase spindles was in the normal range as seen in WT cells (Fig. 5b).

We further analysed the effect of tubacin on Cdc42 activity of cell embedded in Matrigel. It turned out that HDAC6 inhibition did not resume Cdc42 activity (Fig. 5c). This result evoked a question whether HDAC6 inhibition allows cells to develop normal apical domain formation independent of Cdc42. To this end, GFP-Cdc42-N17, a dominant-negative mutant, was expressed in control MDCK cells to address this question. As expected, inactivation of Cdc42 impairs normal apical domain formation. Interestingly, treatment of these cells with tubacin was also capable of reversing the defect to form single apical domain formation in 2-day cysts (Fig. 5d). Given that site-specific activation of aPKC by Cdc42/Par6/aPKC and Par3/aPKC pathway are essential for apical initiation and proper spindle orientation, we treated control MDCK cells with aPKC inhibitor, a pseudosubstrate^47^. As expected, their metaphase spindles were tilted to an average of 30°. Tubacin co-treatment rescued mitotic spindle to near 90° angle towards the centre of cyst (Fig. 5e). As a result, these cells developed normal lumen with single apical domain (Supplementary Fig. 6). Clearly, inhibition of HDAC6 overcomes the loss of aPKC activity to reorient mitotic spindle properly. Thus, the restoration of normal epithelial lumenogenesis by suppressing HDAC6 function is independent of Cdc42 and aPKC activation.

### Concurrent reduction in ERK/myosin II in lumen organization

**Figure 6:**
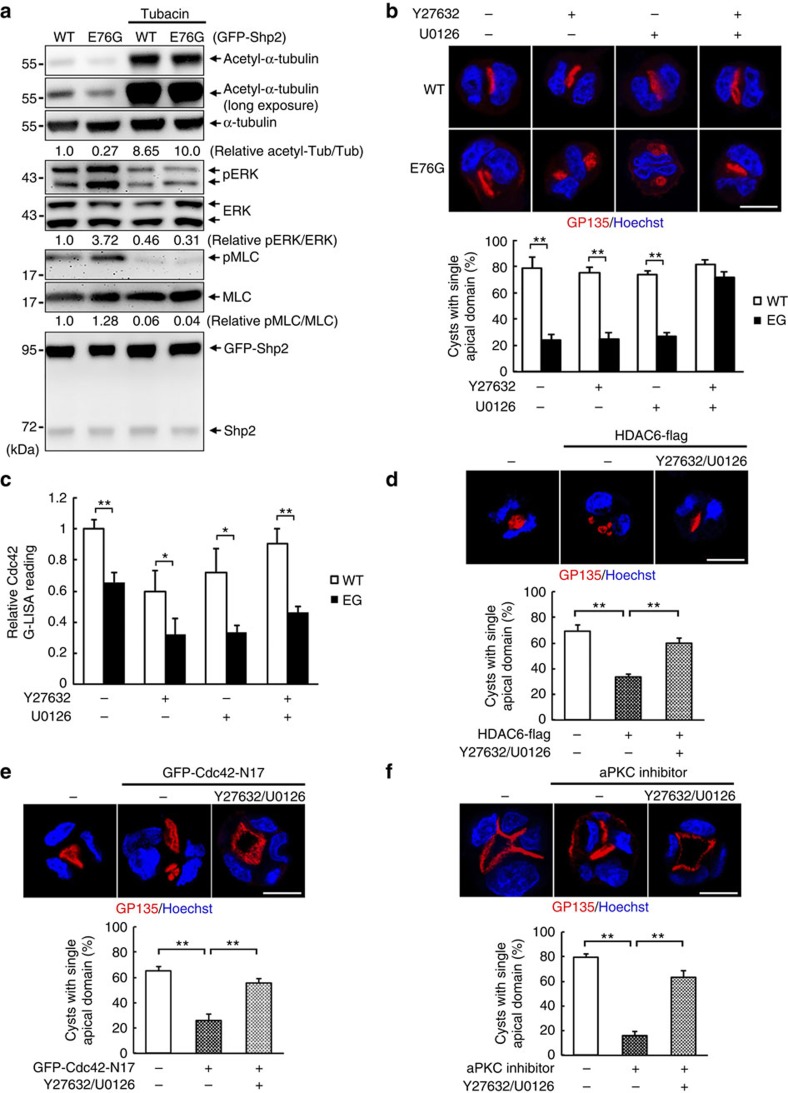
The involvement of ERK/Myosin II in restoring normal lumenogenesis by HDAC6 inhibition. Shp2-WT and E76G cells were embedded in Matrigel for 2 days. (**a**) Western blot analysis of 2-day cysts that had been daily treated with tubacin (1 μM). (**b**) Cyst cultures were treated with Y27632 (10 μM), U0126 (10 μM) or their combination for 2 days. Cysts were extracted for IF staining with antibodies as indicated, scale bar, 10 μm. The percentage of cysts with single apical domain is shown below (323–381 cysts were counted in each experiment, *n*=3). Data are presented as mean±s.d. from three independent experiments; two-tailed unpaired Student's *t*-test. **P<0.01. (**c**) The cysts were harvested for Cdc42 activity by G-LISA assay. (**d**) Control MDCK cells were transfected with the vector GFP-C1 in the presence of control vector or HDAC6-Flag as indicated (amount of DNA ratio at 1:3). These cells were embedded in Matrigel and treated with Y27632/U0126. Cysts were extracted and fixed for IF staining with GP135. Percentage of cysts with single apical domain (158–163 green-positive cysts were counted in each experiment, *n*=3) is shown below and analysed by two-tailed unpaired Student's *t*-test. **P<0.01. (**e**) Control MDCK cells transfected with the vector GFP-C1 or GFP-Cdc42-N17 were embedded in Matrigel and treated without and with Y27632/ U0126. Cysts were extracted and stained with GP135 (red) and Hoechst (blue). The bottom panel shows the percentage of cysts with single apical domain (104–117 green positive were counted in each experiment, *n*=3). Statistical significance was analysed by two-tailed unpaired Student's *t*-test. **P<0.01. (**f**) Control MDCK cells were treated with of aPKC inhibitor (20 μM) without and with Y27632/U0126. The percentage of cysts with single apical domain is shown below (346–393 cysts were counted in each experiment, *n*=3). Values are mean±s.d. from three independent experiments. **P<0.01 based on two-tailed unpaired Student's *t*-test.

We also performed the western blot analysis of 3D cysts. Like HDAC6 depletion, tubacin treatment also increased acetyl-α-tubulin and reduced phosphorylation of both ERK1/2 and MLC (Fig. 6a). By measuring the cell number in each cyst after 3 days of 3D cultures, we found more cells in E76G than WT Shp2 cysts, indicating that oncogenic Shp2 facilitates cell growth in the cyst structure (Supplementary Fig. 7A). Tubacin treatment reduced the cell number and Ki67 staining, a marker of proliferation, in each Shp2-E76G cyst to the level similar to WT Shp2 cyst, suggesting that HDAC6 inhibition resets the normal growth in 3D culture (Supplementary Fig. 7B).

Next, we treated cells with the inhibitor of MEK1/2 and ROCK, U0126 (ref. 48) and Y27632 (ref. 39), respectively, to test whether suppression of ERK and ROCK-mediated MLC phosphorylation^49^ contribute to the rescued effect by tubacin. Aberration in apical initiation in two-cell stage of E76G cysts was reverted to a single apical domain by simultaneous treatment of U0126 and Y27632 (Fig. 6b). The individual treatments had no effect on restoring a single apical domain in two-cell stage of Shp2E76 cells. Consistently, co-treatment with U0126 and Y27632, but not the individual treatment, rescued Shp2-E76G cells to expand normal lumen formation in a 7-day culture (Supplementary Fig. 8). Of note, Cdc42 activity in Shp2-E76G cyst was not increased by U0126 and Y27632 treatment (Fig. 6c). Thus, concurrent suppression of ERK1/2 and myosin II has the same effect as HDAC6 inhibition in preventing lumen defect without restoring Cdc42 activity. Overexpression of HDAC6 that caused abnormal apical domain defect in control MDCK cells was also reversed by the combinatorial treatment of U0126 and Y27632 (Fig. 6d). Based on these results, we hypothesized that hyperactivation of Shp2 impairs Tuba-Cdc42 signal and decreases microtubule acetylation. HDAC6 inhibition highly promotes microtubule acetylation to make a double play in suppressing myosin II-mediated contractility and hyperactivation of ERK1/2 simultaneously. This cellular context overcomes oncogenic Shp2-E76G-induced Cdc42 defect to enable normal growth and lumenogenesis in 3D culture. We further tested the effect of co-suppression of MEK and ROCK on reversing aberration of apical domain in control MDCK cells expressing Cdc42-N17 or treated with aPKC inhibitor. Like tubacin, this co-treatment rescued normal apical domain formation (Fig. 6e,f).

### Shp2 represses Cdc42 in the lumen defect of cancer cells

**Figure 7:**
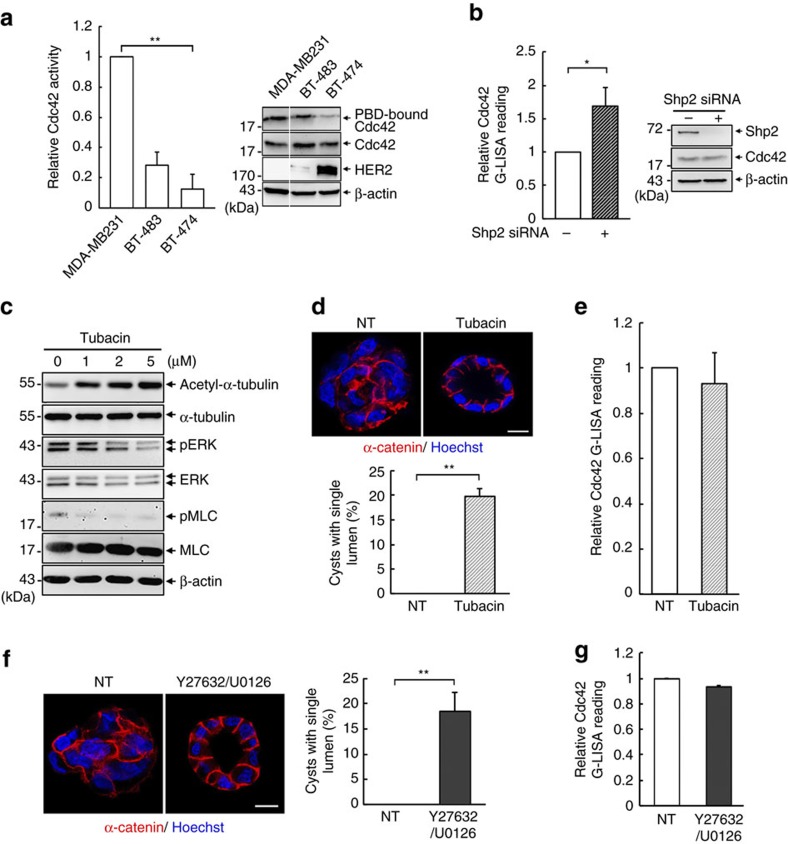
Shp2 and HDAC6 influence epithelial organization of HER2-positive breast cancer cells. (**a**) Various breast cancer cell lines were lysed and subjected for Cdc42 activity assay by GST-PBD pull-down. Relative endogenous Cdc42 activity from three independent experiments is shown. Statistical significance was analysed by two-tailed unpaired Student's *t*-test. *P<0.05, **P<0.01. (**b**) BT474 cells were transfected with Shp2 siRNA, and then harvested for Cdc42 activity assay. Data are presented as mean±s.d.; statistical significance was analysed by 2-tailed unpaired Student's *t*-test. *P<0.05, (**c**) BT474 cells were daily treated with Tubacin at the indicated concentration for 2 days, and harvested for western blot analysis. (**d**) Cells were cultured in Matrigel with daily treatment of tubacin (5 μM) for 2 weeks. These cysts were extracted for IF staining with anti-β-catenin (red) and Hoechst (blue). Percentage of cysts with single lumen (300 cysts were counted, *n*=3)Were analysed by two-tailed unpaired Student's *t*-test. **P<0.01. (**e**) Cdc42 activity was analysed in 2 days of cysts. (**f**) BT474 cells treated with or without Y27632 (10 μM) and U0126 (10 μM) for 2 weeks were extracted for IF staining. Statistical significance was analysed by two-tailed unpaired Student's *t*-test. **P<0.01. (**g**) Cdc42 activity in 2-day cysts by G-LISA assay.

Next, we examined these findings relevant to cancer. Shp2 deregulation by oncogenic HER2 that causes epithelial disorganization has been documented in breast cancer^20^,^21^,^22^. We compared Cdc42 activity in different breast cancer cell lines to assess the relationship of Cdc42 activity with HER2 expression. The result showed that BT474 cells with strongest HER2-postive signal had the lowest Cdc42 activity (Fig. 7a). Knockdown of Shp2 in BT474 cells increased Cdc42 activity (Fig. 7b), supporting the negative regulation of Cdc42 by Shp2. This might explain why knockdown of Shp2 is able to restore normal lumen formation in BT474 cells^20^,^21^,^22^. We then tested the effect of tubacin treatment on cyst formation of BT474 cells in 3D cultures. BT474 cells developed irregular cysts without central lumen. Similar to Shp2-E76G MDCK cells, treatment of BT474 cells with tubacin reduced phosphorylation of ERK1/2 and MLC, and promoted normal apical domain without increasing Cdc42 activity (Fig. 7c–e). The combination of U0126 with Y27632 also caused a proportion of cysts with a central lumen (Fig. 7f). Again, Cdc42 activity in these cysts was not increased by the treatment (Fig. 7g). Thus, suppressing HDAC6 or MEK/ROCK together promotes normal epithelial organization in HER2-positive BT474 cancer cells that have low Cdc42 function.

## Discussion

Epithelial morphogenesis is important for cell development and differentiation. Disruption of this process causes various diseases and is involved in tumorigenesis^24^,^25^,^26^. Several epithelial polarity regulators have been shown to have tumor suppressor function and can cooperate with oncogenes to drive metastasis.

In this study, we highlight the disruption of lumenogenesis by oncogenic function of Shp2 through decreasing Cdc42 activity. Our mechanistic investigation reveals that tyrosine 430/515 phosphorylation of Tuba positively regulates its GEF function in Cdc42 activation. Aberrant Shp2 activation reduces tyrosine phosphorylation of Tuba to decrease Cdc42 activity. Therefore, oncogenic Shp2 impairs normal lumenogenesis via repressing Cdc42 activation.

Tuba is a scaffold protein that links dynamin with actin-regulating proteins^31^. It is a specific Cdc42 guanine exchange factor in Rab11a-mediated transcytotic pathway essential for apical vesicle transport, forming the apical membrane domain^30^. Knockdown of Tuba has been shown to cause multiple lumens in epithelial cysts^30^,^32^. This is similar to the phenotype observed in MDCK cells with Shp2-E76G expression. Since unphosphorylated Tuba had low GEF activity for Cdc42, Tuba is one of GEF's positively regulated by tyrosine phosphorylation. Given phosophorylated Tuba as a substrate of Shp2, aberrant activation of Shp2 that decreases Tuba phosphorylation reduces Cdc42 activity, thereby resulting in multi-lumen as seen in Tuba depleted cells.

GP135 vesicle transport from basolateral surface to apical initiation site requires Cdc42 and the exocyst-dependent Rab11a/Rab8 vesicle docking. The binding of Cdc42 to Par6/Crb3 is required for aPKC localization to the forming apical membrane at two-cell stage in 3D culture^24^,^26^,^30^. In this process, interaction of the exocyst with Par3-aPKC is essential for the vesicle delivery^26^. Subsequent to apical domain formation, Cdc42/Par6/aPKC is essential for the spindle position for pre-apical patch formation and lumen expansion. Phosphorylation of Pin1 by aPKC causes 14-3-3 binding to exclude Pin1 from the apical cortex, thus preventing spindle misorientation^50^. In this study, HDAC6 inhibition restores normal apical domain formation and prevents the mitotic spindle misorientation in Shp2-E76G cells without increasing global Cdc42 activity. Importantly, in control MDCK cells, HDAC6 inhibition not only reverses Cdc42-N17-induced multiple apical GP135 vacuole formation, but also gives proper spindle orientation when aPKC is inhibited. These data imply that HDAC6 inhibition offers a situation to allow apical targeting and reorient spindle in the epithelial cells without the need for Cdc42 and aPKC. This evokes the question why inhibition of HDAC6 is able to replace the role of Cdc42/Par6/Par3/aPKC signalling in establishing a single apical domain at two-cell stage and the proper mitotic spindle positioning in cell division during luminogenesis. In Shp2-E76G cells, ERK and ROCK are hyperactive because of Shp2-mediated Ras signalling^13^ and dephosphorylation of ROCK^38^. Intriguingly, we found that HDAC6 inhibition in Shp2-E76G cells simultaneously suppressed ROCK-mediated MLC phosphorylation and ERK activation. Conversely, overexpression of HDAC6 leads to hyper-activation of these two events, indicating both ERK and myosin II activity are co-regulated by HDAC6. Like HDAC6 inhibition, co-suppression of ROCK and ERK restores normal apical initiation and spindle orientation in Shp2-E76G or aPKC-inhibited MDCK cells. It has been shown that reduced ROCK activity is required for centrosomal positioning^51^,^52^ and GP135 endocytosis from cell periphery^26^. Therefore, hyperactivation of ROCKII in E76G cells contributes to polarity defect. Since Cdc42 is still defective, Y27632 treatment on its own, as expected, has on effect on multi-lumen formation in Shp2-E76G cells. So, the unknown issue is the function of ERK1/2 in relation to apical vesicle trafficking and spindle orientation. In particular, co-suppression of ERK and ROCK also prevents apical lumen defect in control MDCK cells with aPKC or Cdc42 inhibition. Therefore, this finding opens a question of how the reduction of myosin II and ERK1/2 cooperate to allow lumen formation without Cdc42 or aPKC activation. One possible scenario is that ERK and ROCK signals form the intrinsic block(s) for normal apical domain formation; in a condition that cells proliferate in a normal rate, Cdc42-dependent apical localization of aPKC is critical for apical domain formation. When cells proliferate slower, the need for Cdc42 or aPKC regulation might be compromised by the loss of these intrinsic blocks to establish an alternative pathway, allowing normal lumen formation.

In BT474, a HER2-positive breast cancer cell line, we also found low Cdc42 activity due to Shp2-mediated repression. Similar to Shp2-E76G MDCK cells, we found that either HDAC6 inhibition alone or co-suppression of ERK and ROCK in BT474 cells promotes their normal epithelial phenotype without increasing Cdc42 activity. It is known that Shp2 deficient mice are lethal^12^,^53^, while HDAC6 knockout mice are viable^54^. Although Shp2 inhibitor is suggested to have potential in cancer therapy, the tumor suppressor function of Shp2 has also been found^55^. The pleiotropic effects of suppressing HDAC6 in preventing aberrant Shp2-induced abnormalities in epithelial growth and morphogenesis suggest the medical implication of HDAC6 inhibitor in managing the developmental defect and diseases involving Shp2 deregulation.

## Methods

### Plasmids and reagents

GFP-Cdc42-WT, -V12 and N17 plasmids were obtained from Dr T. S. Jou. HA-Tuba (*Homo sapiens*) plasmid was from Addgene. Primary antibodies clones and final dilution were as followed: anti-Cdc42 (sc-87, 1:1,000), anti-ERK2 (sc-154, 1:4,000) and anti-SH-PTP2 (sc-7384, 1:4,000) antibodies were from Santa Cruz, anti-E-cadherin (13–1900, 1:2,000) and anti-ZO-1 (33–9100, 1:2,000) antibodies were from Zymed, anti-β-actin (A5441, 1:4,000), anti-β-tubulin (T4026, 1:4,000), anti-α-catenin (C2081, WB:1:2,000, IF: 1:200), anti-MLC (M4401, 1:1,000), anti-α-tubulin (T6199, WB:1:4,000, IF:1:200), and anti-acetyl-α-tubulin (T7451, 1:4,000) antibodies were from Sigma-Aldrich, anti-phospho-p44/42 MAPK (T202/Y204) (4370, 1:4,000), and anti-phospho-MLC2 (T18/S19) (3674S, 1:1,000) antibodies were form Cell Signaling Technology, anti-β-catenin (610154, WB:1:2,000, IF: 1:200) antibody was from BD Biosciences. Anti-HA antibody (GTX115044, 1:1,000) was from Genetex. Anti-phosphotyrosine, clone 4G10 (05–321, 1:5,000) was from Millipore. Anti-HER2 and anti-GP135 antibodies were provided by J. Y. Shew (Genomics Research Center, Academia Sinica) and Dr T. S. Jou (National Taiwan University College of Medicine), respectively. Anti-pS1366-ROKII antibody was produced by our laboratory^37^. Tubacin were purchased from Calbiochem. Matrigel was from BD Biosciences. The smart pool siRNA targeting human Shp2 was obtained from Thermo Fisher Scientific. HDAC6 siRNA (sense: 5′-GUUUGAUGAGCAACUAAAUdTdT-3′) and Tuba siRNA (sense: 5′-CAUAAUACACGUGUAUUAUdTdT-3′) were obtained from Sigma-Aldrich.

### Cell culture

MDCK cells were obtained from American Type Culture Collection. MDCK stably expressing GFP-Shp2-WT and -E76G cells were generated by transfection their expression vector carrying Zeocin resistance gene for selection as previously described^35^. Cell lines were maintained in DMEM supplemented with 10% FBS and 20 ng ml^−1^ of doxycycline in a humidified atmosphere of 5% CO_2_/95% air at 37 °C. Breast cancer cell lines, MDA-MB231, BT474 and BT483, were from American Type Culture Collection and cultured in DMEM/F12 medium supplemented with 10% heat-inactivated FBS.

### 3D culture

Matrigel was added to a 24-well dish and plated in incubator to allow 20 min for the Matrigel to solidify. Suspension of MDCK cells (5 × 10^4^ ml^−1^) were mixed in Matrigel (BD Biosciences) and grown in culture medium containing 2% Matrigel^55^. After 2 or 7 days, ice-cold PBS–EDTA was added to detach Matrigel from bottom and shaken gently for 15 min at 4 °C. The solution was transferred to microtube and gently shaken until Matrigel has been dissolved completely. Cysts were centrifuged at 2,000 r.p.m. for 1 min and applied onto a poly-L-lysine-coated coverslips for immunofluorescence analysis.

### Spindle orientation

MDCK cells were embedded in Matrigel and cultured in eight-well chamber slide for 24 h. To enrich the mitotic population, cysts were treated with 10 μM of RO-3306 to synchronize at G2/M phase, After 16 h, cysts were released into normal culture medium for 40–50 min and fixed by paraformaldehyde. Cysts were stained with α-tubulin and Hoechst. To measure the spindle orientation, we marked the spindle pole and the centre of the cysts. We drew a thin line connecting centre of cysts and midpoint of spindle axis. The angle between the thin line and spindle axis was calculated.

### Endogenous Cdc42 activity assay

Endogenous Cdc42 activity was determined by GST-PBD pull-down assay. Briefly, the lysates were incubated at 4 °C for 1 h with GST-PBD beads and the amounts of total and active Cdc42 were detected by western blot. G-LISA Cdc42 Activation Assay Biochem Kit (Cytoskeleton, BK127) were used to measure levels of activated Cdc42 in 3D cysts according to the manufacturer's instructions.

### GEF activity assay of Tuba

HEK293T cells were transfected with HA-Tuba and lysed in lysis buffer containing 50 mM HEPES, pH 7.5, 1% Triton X-100, 150 mM NaCl, 20 mM NaF, 1 mM Na_3_VO_4_, 1 mM DTT, 1 mM phenylmethylsulphonyl fluoride and protease inhibitor cocktail. The cell lysates were precleared by incubation with GST beads for 10 min at 4 °C, after which the supernatants were collected for pull-down by GST-Cdc42-G15A beads. Cdc42-G15A is free of nucleotide binding and has a high affinity for binding with active GEFs. The precipitated HA-Tuba was analysed by western blot using HA antibody.

### *In vitro* dephosphorylation assay

HA-Tuba protein was immunoprecipitated from pervanadate-treated HEK293T cells and then incubated with or without immune-purified Flag-Shp2-E76G protein eluted from M2 beads in a reaction buffer containing 25 mM of Tris, pH 7.0, 50 mM NaCl, 2 mM EDTA, 5 mM DTT and 0.1 mg ml^−1^ of BSA at 30 °C for 30 min, The reaction was stopped by the addition of Laemmli buffer and boiled at 95 °C for 10 min.

### Western blot analysis

Cells were washed with ice-cold PBS three times and lysed in a buffer containing 50 mM Tris-HCl, pH 7.2, 150 mM NaCl, 10 mM MgCl_2_, 1% Triton X-100, 0.5% sodium deoxycholate, 0.1% SDS, 50 mM NaF, 2 mM Na_3_VO_4_ and protease and phosphatase inhibitor cocktail. Cell lysates were resolved by SDS–PAGE for immunoblotting with the indicated antibodies and then detected with peroxidase-conjugated secondary antibodies and chemiluminescent (ECL) reagent. Uncropped western blots are shown in Supplementary Fig. 9.

### Immunofluorescence staining and image analysis

Cells and cysts plated on glass coverslips or eight-well chamber slide were washed with PBS and fixed with 4% paraformaldehyde for 30 min, followed by permeabilization with 0.3% of TritonX-100 and blocked with 5.5% of normal goat serum before staining with primary antibodies. Then, cells were washed and incubated with TRITC-, FITC- or Alexa-633-conjugated secondary antibodies for 1 h. F-actin was detected by staining with rhodamine-phalloidin. Images were obtained by the Zeiss LSM 510 and LSM 700 confocal laser scanning microscope. ZEN 2009 Light Edition and AxioVision Rel. 4.8 (Carl Zeiss) software were used for image quantification and analysis.

## Additional information

**How to cite this article:** Tien, S.-C. *et al.* The Shp2-induced epithelial disorganization defect is reversed by HDAC6 inhibition independent of Cdc42. *Nat. Commun.* 7:10420 doi: 10.1038/ncomms10420 (2016).

## Supplementary Material

Supplementary Information Supplementary Figures 1-9 and Supplementary Methods.

## References

[b1] Neel B. G., Gu H. & Pao L. The 'Shp'ing news: SH2 domain-containing tyrosine phosphatases in cell signaling. Trends Biochem. Sci. 28, 284–293 (2003). 12826400 10.1016/S0968-0004(03)00091-4

[b2] Zheng H., Alter S. & Qu C. K. SHP-2 tyrosine phosphatase in human diseases. Int. J. Clin. Exp. Med. 2, 17–25 (2009). 19436828 PMC2680053

[b3] Huang W. Q. *et al.* Structure, function, and pathogenesis of SHP2 in developmental disorders and tumorigenesis. Curr. Cancer Drug Targets 14, 567–588 (2014). 25039348 10.2174/1568009614666140717105001

[b4] Li S. *et al.* Cytoplasmic tyrosine phosphatase Shp2 coordinates hepatic regulation of bile acid and FGF15/19 signaling to repress bile acid synthesis. Cell Metab. 20, 320–332 (2014). 24981838 10.1016/j.cmet.2014.05.020 PMC4365973

[b5] Hof P., Pluskey S., Dhe-Paganon S., Eck M. J. & Shoelson S. E. Crystal structure of the tyrosine phosphatase SHP-2. Cell 92, 441–450 (1998). 9491886 10.1016/s0092-8674(00)80938-1

[b6] Bentires-Alj M. *et al.* Activating mutations of the noonan syndrome-associated SHP2/PTPN11 gene in human solid tumors and adult acute myelogenous leukemia. Cancer Res. 64, 8816–8820 (2004). 15604238 10.1158/0008-5472.CAN-04-1923

[b7] Araki T. *et al.* Mouse model of Noonan syndrome reveals cell type- and gene dosage-dependent effects of Ptpn11 mutation. Nat. Med. 10, 849–857 (2004). 15273746 10.1038/nm1084

[b8] Tartaglia M. *et al.* Somatic mutations in PTPN11 in juvenile myelomonocytic leukemia, myelodysplastic syndromes and acute myeloid leukemia. Nat. Genet. 34, 148–150 (2003). 12717436 10.1038/ng1156

[b9] Tartaglia M. *et al.* Diversity and functional consequences of germline and somatic PTPN11 mutations in human disease. Am. J. Hum. Genet. 78, 279–290 (2006). 16358218 10.1086/499925 PMC1380235

[b10] Mohi M. G. & Neel B. G. The role of Shp2 (PTPN11) in cancer. Curr. Opin. Genet. Dev. 17, 23–30 (2007). 17227708 10.1016/j.gde.2006.12.011

[b11] Bentires-Alj M. *et al.* A role for the scaffolding adapter GAB2 in breast cancer. Nat. Med. 12, 114–121 (2006). 16369543 10.1038/nm1341

[b12] Saxton T. M. *et al.* Abnormal mesoderm patterning in mouse embryos mutant for the SH2 tyrosine phosphatase Shp-2. EMBO J. 16, 2352–2364 (1997). 9171349 10.1093/emboj/16.9.2352 PMC1169836

[b13] Krenz M. *et al.* Role of ERK1/2 signalling in congenital valve malformations in Noonan syndrome. Proc. Natl Acad. Sci. USA 105, 18930–18935 (2008). 19017799 10.1073/pnas.0806556105 PMC2596231

[b14] Wu X. *et al.* MEK-ERK pathway modulation ameliorates disease phenotypes in a mouse model of Noonan syndrome associated with the Raf1(L613V) mutation. J. Clin. Invest. 121, 1009–1025 (2011). 21339642 10.1172/JCI44929 PMC3049402

[b15] De Rocca Serra-Nedelec A. *et al.* Noonan syndrome-causing SHP2 mutants inhibit insulin-like growth factor 1 release via growth hormone-induced ERK hyperactivation, which contributes to short stature. Proc. Natl Acad. Sci. USA 109, 4257–4262 (2012). 22371576 10.1073/pnas.1119803109 PMC3306697

[b16] Dance M., Montagner A., Salles J. P., Yart A. & Raynal P. The molecular functions of Shp2 in the Ras/Mitogen-activated protein kinase (ERK1/2) pathway. Cell Signal. 20, 453–459 (2008). 17993263 10.1016/j.cellsig.2007.10.002

[b17] Maroun C. R., Naujokas M. A., Holgado-Madruga M., Wong A. J. & Park M. The tyrosine phosphatase SHP-2 is required for sustained activation of extracellular signal-regulated kinase and epithelial morphogenesis downstream from the met receptor tyrosine kinase. Mol. Cell. Biol. 20, 8513–8525 (2000). 11046147 10.1128/mcb.20.22.8513-8525.2000 PMC102157

[b18] Saadat I. *et al.* Helicobacter pylori CagA targets PAR1/MARK kinase to disrupt epithelial cell polarity. Nature 447, 330–333 (2007). 17507984 10.1038/nature05765

[b19] Higashi H. *et al.* SHP-2 tyrosine phosphatase as an intracellular target of *Helicobacter pylori* CagA protein. Science 295, 683–686 (2002). 11743164 10.1126/science.1067147

[b20] Zhou X. D. & Agazie Y. M. Inhibition of SHP2 leads to mesenchymal to epithelial transition in breast cancer cells. Cell Death Differ. 15, 988–996 (2008). 18421299 10.1038/cdd.2008.54

[b21] Zhou X. & Agazie Y. M. Molecular mechanism for SHP2 in promoting HER2-induced signaling and transformation. J. Biol. Chem. 284, 12226–12234 (2009). 19261604 10.1074/jbc.M900020200 PMC2673291

[b22] Aceto N. *et al.* Tyrosine phosphatase SHP2 promotes breast cancer progression and maintains tumor-initiating cells via activation of key transcription factors and a positive feedback signaling loop. Nat. Med. 18, 529–537 (2012). 22388088 10.1038/nm.2645

[b23] Thiery J. P., Acloque H., Huang R. Y. & Nieto M. A. Epithelial-mesenchymal transitions in development and disease. Cell 139, 871–890 (2009). 19945376 10.1016/j.cell.2009.11.007

[b24] Datta A., Bryant D. M. & Mostov K. E. Molecular regulation of lumen morphogenesis. Curr. Biol. 21, R126–R136 (2011). 21300279 10.1016/j.cub.2010.12.003 PMC3771703

[b25] McCaffrey L. M., Montalbano J., Mihai C. & Macara I. G. Loss of the Par3 polarity protein promotes breast tumorigenesis and metastasis. Cancer Cell 22, 601–614 (2012). 23153534 10.1016/j.ccr.2012.10.003 PMC3500525

[b26] Overeem A. W., Bryant D. M. & van I. S. C. Mechanisms of apical-basal axis orientation and epithelial lumen positioning. Trends Cell Biol. 25, 476–485 (2015). 25941134 10.1016/j.tcb.2015.04.002

[b27] Apodaca G., Gallo L. I. & Bryant D. M. Role of membrane traffic in the generation of epithelial cell asymmetry. Nat. Cell Biol. 14, 1235–1243 (2012). 23196841 10.1038/ncb2635 PMC3771702

[b28] Horikoshi Y. *et al.* Interaction between PAR-3 and the aPKC-PAR-6 complex is indispensable for apical domain development of epithelial cells. J. Cell Sci. 122, 1595–1606 (2009). 19401335 10.1242/jcs.043174

[b29] Goldstein B. & Macara I. G. The PAR proteins: fundamental players in animal cell polarization. Dev. Cell 13, 609–622 (2007). 17981131 10.1016/j.devcel.2007.10.007 PMC2964935

[b30] Bryant D. M. *et al.* A molecular network for de novo generation of the apical surface and lumen. Nat. Cell Biol. 12, 1035–1045 (2010). 20890297 10.1038/ncb2106 PMC2975675

[b31] Cestra G., Kwiatkowski A., Salazar M., Gertler F. & De Camilli P. Tuba, a GEF for CDC42, links dynamin to actin regulatory proteins. Methods Enzymol. 404, 537–545 (2005). 16413298 10.1016/S0076-6879(05)04047-4

[b32] Qin Y., Meisen W. H., Hao Y. & Macara I. G. Tuba, a Cdc42 GEF, is required for polarized spindle orientation during epithelial cyst formation. J. Cell Biol. 189, 661–669 (2010). 20479467 10.1083/jcb.201002097 PMC2872902

[b33] Hubbert C. *et al.* HDAC6 is a microtubule-associated deacetylase. Nature 417, 455–458 (2002). 12024216 10.1038/417455a

[b34] Gao Y. S., Hubbert C. C. & Yao T. P. The microtubule-associated histone deacetylase 6 (HDAC6) regulates epidermal growth factor receptor (EGFR) endocytic trafficking and degradation. J. Biol. Chem. 285, 11219–11226 (2010). 20133936 10.1074/jbc.M109.042754 PMC2856999

[b35] Tien S. C. & Chang Z. F. Oncogenic Shp2 disturbs microtubule regulation to cause HDAC6-dependent ERK hyperactivation. Oncogene 33, 2938–2946 (2014). 23770849 10.1038/onc.2013.241

[b36] Joo E. E. & Yamada K. M. MYPT1 regulates contractility and microtubule acetylation to modulate integrin adhesions and matrix assembly. Nat. Commun. 5, 3510 (2014). 24667306 10.1038/ncomms4510 PMC4190669

[b37] Chuang H. H. *et al.* ROCKII serine 1366 phosphorylation reflects the activation status. Biochem. J. 443, 145–151 (2012). 22273145 10.1042/BJ20111839

[b38] Lee H. H. & Chang Z. F. Regulation of RhoA-dependent ROCKII activation by Shp2. J. Cell Biol. 181, 999–1012 (2008). 18559669 10.1083/jcb.200710187 PMC2426933

[b39] Ishizaki T. *et al.* Pharmacological properties of Y-27632, a specific inhibitor of rho-associated kinases. Mol. Pharmacol. 57, 976–983 (2000). 10779382

[b40] Garcia-Mata R. *et al.* Analysis of activated GAPs and GEFs in cell lysates. Methods Enzymol. 406, 425–437 (2006). 16472675 10.1016/S0076-6879(06)06031-9

[b41] Bai Y. *et al.* Phosphoproteomics identifies driver tyrosine kinases in sarcoma cell lines and tumors. Cancer Res. 72, 2501–2511 (2012). 22461510 10.1158/0008-5472.CAN-11-3015 PMC4641440

[b42] Iliuk A. B., Martin V. A., Alicie B. M., Geahlen R. L. & Tao W. A. In-depth analyses of kinase-dependent tyrosine phosphoproteomes based on metal ion-functionalized soluble nanopolymers. Mol. Cell. Proteomics. 9, 2162–2172 (2010). 20562096 10.1074/mcp.M110.000091 PMC2953913

[b43] Hornbeck P. V. *et al.* PhosphoSitePlus: a comprehensive resource for investigating the structure and function of experimentally determined post-translational modifications in man and mouse. Nucleic Acids Res. 40, D261–D270 (2012). 22135298 10.1093/nar/gkr1122 PMC3245126

[b44] Haggarty S. J., Koeller K. M., Wong J. C., Grozinger C. M. & Schreiber S. L. Domain-selective small-molecule inhibitor of histone deacetylase 6 (HDAC6)-mediated tubulin deacetylation. Proc. Natl Acad. Sci. USA 100, 4389–4394 (2003). 12677000 10.1073/pnas.0430973100 PMC153564

[b45] Durgan J., Kaji N., Jin D. & Hall A. Par6B and atypical PKC regulate mitotic spindle orientation during epithelial morphogenesis. J. Biol. Chem. 286, 12461–12474 (2011). 21300793 10.1074/jbc.M110.174235 PMC3069449

[b46] Jaffe A. B., Kaji N., Durgan J. & Hall A. Cdc42 controls spindle orientation to position the apical surface during epithelial morphogenesis. J. Cell Biol. 183, 625–633 (2008). 19001128 10.1083/jcb.200807121 PMC2582895

[b47] Eichholtz T., de Bont D. B., de Widt J., Liskamp R. M. & Ploegh H. L. A myristoylated pseudosubstrate peptide, a novel protein kinase C inhibitor. J. Biol. Chem. 268, 1982–1986 (1993). 8420972

[b48] Favata M. F. *et al.* Identification of a novel inhibitor of mitogen-activated protein kinase kinase. J. Biol. Chem. 273, 18623–18632 (1998). 9660836 10.1074/jbc.273.29.18623

[b49] Amano M. *et al.* Phosphorylation and activation of myosin by Rho-associated kinase (Rho-kinase). J. Biol. Chem. 271, 20246–20249 (1996). 8702756 10.1074/jbc.271.34.20246

[b50] Hao Y. *et al.* Par3 controls epithelial spindle orientation by aPKC-mediated phosphorylation of apical Pins. Curr. Biol. 20, 1809–1818 (2010). 20933426 10.1016/j.cub.2010.09.032 PMC2963683

[b51] Rodriguez-Fraticelli A. E., Auzan M., Alonso M. A., Bornens M. & Martin-Belmonte F. Cell confinement controls centrosome positioning and lumen initiation during epithelial morphogenesis. J. Cell Biol. 198, 1011–1023 (2012). 22965908 10.1083/jcb.201203075 PMC3444774

[b52] Rodriguez-Fraticelli A. E. & Martin-Belmonte F. Mechanical control of epithelial lumen formation. Small GTPases 4, 136–140 (2013). 23511851 10.4161/sgtp.24303 PMC3747256

[b53] Yang W. *et al.* An Shp2/SFK/Ras/Erk signalling pathway controls trophoblast stem cell survival. Dev. Cell 10, 317–327 (2006). 16516835 10.1016/j.devcel.2006.01.002

[b54] Zhang Y. *et al.* Mice lacking histone deacetylase 6 have hyperacetylated tubulin but are viable and develop normally. Mol. Cell Biol. 28, 1688–1701 (2008). 18180281 10.1128/MCB.01154-06 PMC2258784

[b55] Bard-Chapeau E. A. *et al.* Ptpn11/Shp2 acts as a tumor suppressor in hepatocellular carcinogenesis. Cancer Cell 19, 629–639 (2011). 21575863 10.1016/j.ccr.2011.03.023 PMC3098128

