# Supplementary material for: The Shp2-induced epithelial disorganization defect is reversed by HDAC6 inhibition independent of Cdc42
Source: Nat Commun. 2016 Jan 19;7:10420. doi: 10.1038/ncomms10420 (PMC4735695; doi:10.1038/ncomms10420)
Supplement: Supplementary Information — Supplementary Figures 1-9 and Supplementary Methods. [file ncomms10420-s1.pdf]

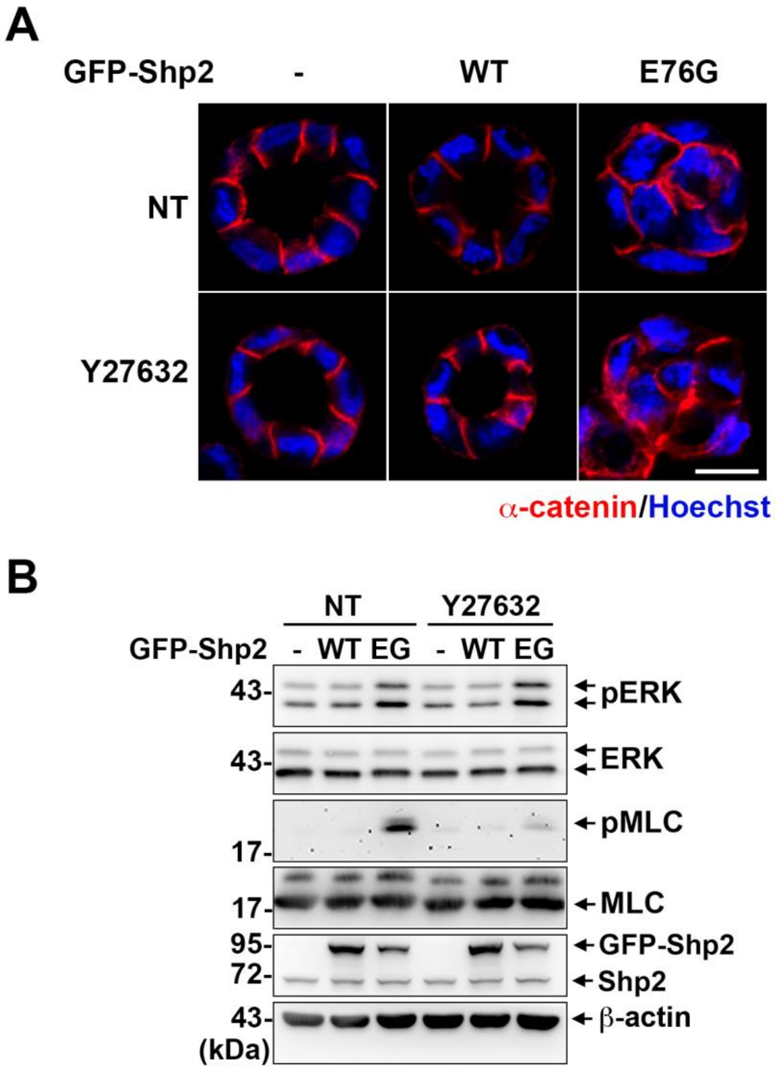

Supplementary Figure 1 Effect of ROCK inhibition on lumen abnormality in MDCK cysts. **(A)** MDCK cells as indicated cultured in Matrigel were treated with and without Y27632 (10 μM) for 1 week. Cysts were extracted for IF staining with α-catenin (Red) and Hoechst (blue). Scale bar, 10 μm. **(B)** These cysts were lysed for Western blot analysis with the indicated antibodies.

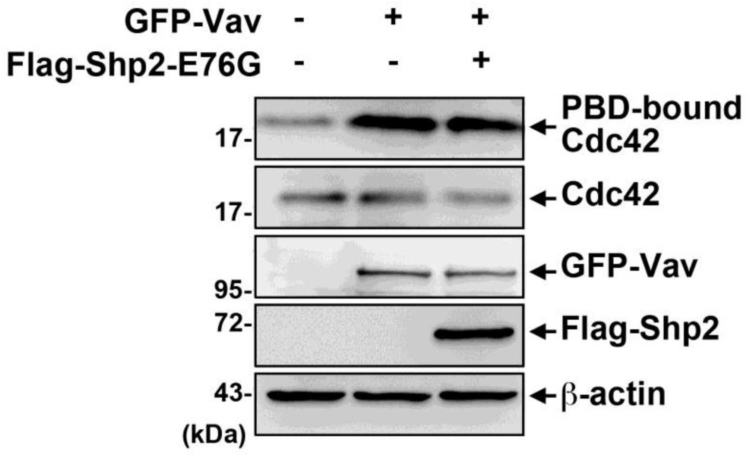

Supplementary Figure 2 Expression of oncogenic Shp2 has no effect on Vav1-induced Cdc42 activation. HEK293T cells were co-transfected with GFP-Vav1 with or without Flag-Shp2 for overnight. Cells were harvested and subjected for endogenous Cdc42 activity assay by GST-PBD pull-down. The total expression levels of Cdc42, GFP-Vav, and Flag-Shp2 were detected by Western blot analysis as indicated.

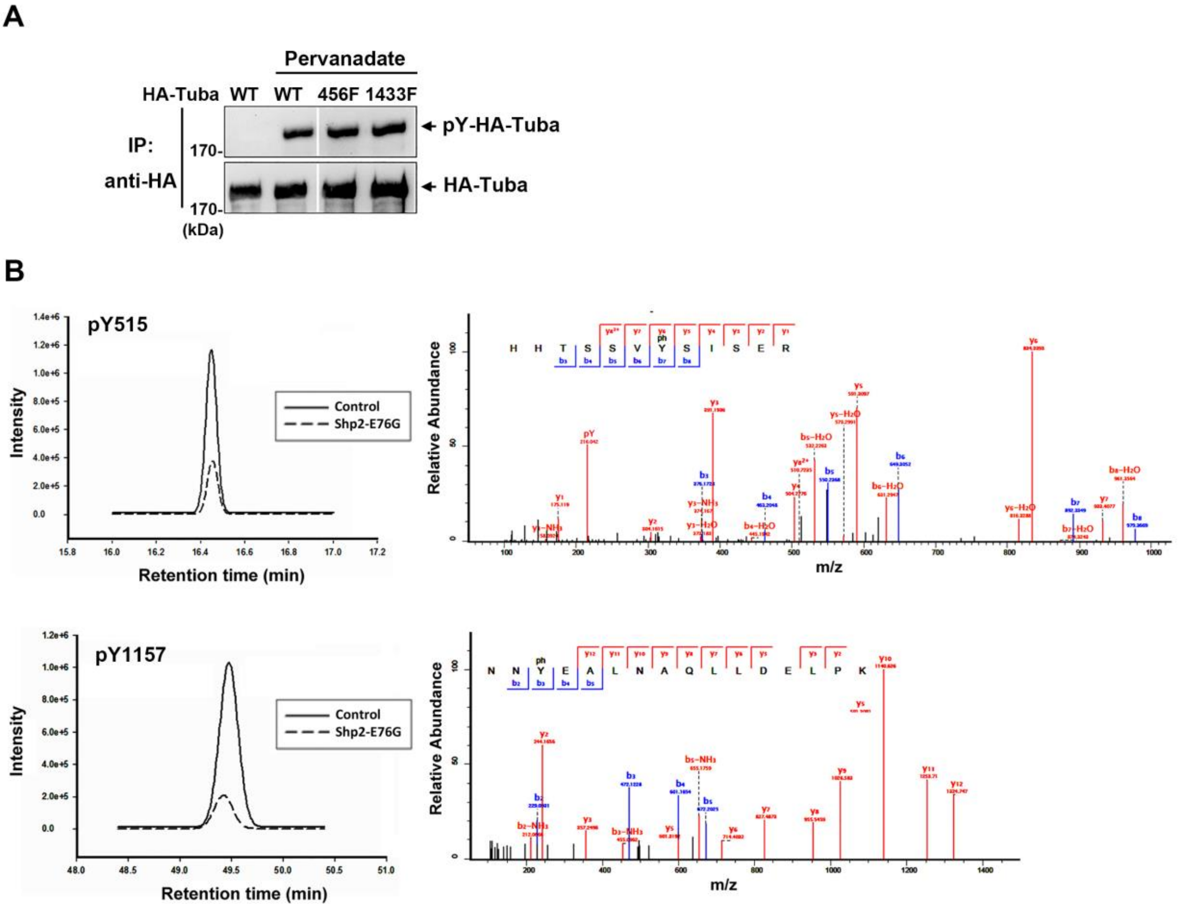

Supplementary Figure 3 Analysis of phosphorylation sites of Tuba.

(A) The expression vector of HA-Tuba carrying WT, Y456F and Y1433F mutation was transfected to HEK293T cells. After 24h, cells were treated with pervanadate (50μM) for 10 min and harvested for Western blot analysis using anti-phosphotyrosine (4G10) antibody. **(B)** HA-Tuba protein was immunoprecipitated from HEK293T cells treated with pervanadate (50μM) for 10 min. Cell lysates were and incubated with purified Flag-Shp2-E76G protein from M2 beads. HA-Tuba protein was separated by SDS PAGE and visualized by Coomassie blue staining. The gel containing HA-Tuba protein was excised for in-gel digestion by trypsin, after which phosphopepides were purified by tip-based IMAC procedures. The eluted peptides were desalted using reversed phase-stageTips for mass spectrometric (MS) analysis. Panel in the left shows the quantitation of phosphopeptide identified by the MS system, which sequence is shown in the right.

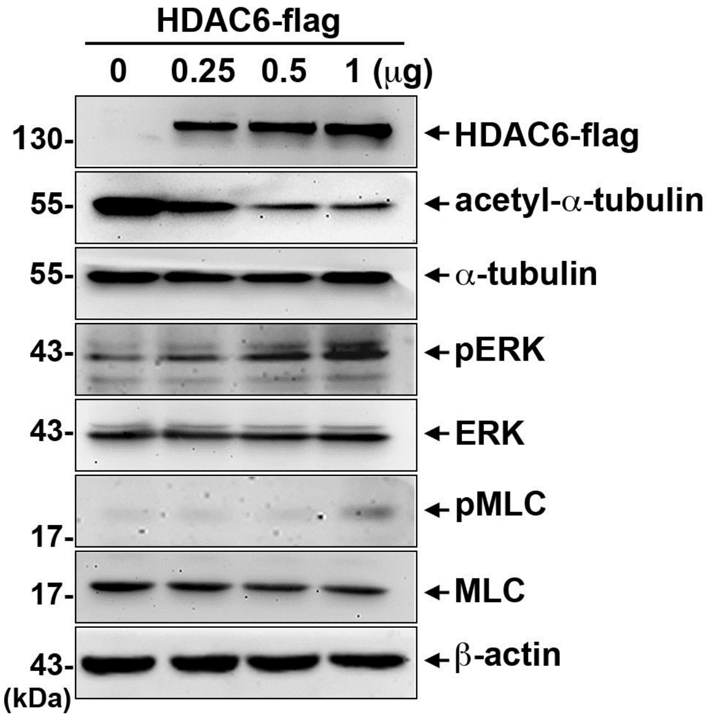

Supplementary Figure 4 The effect of HDAC6 overexpression on phosphorylation of ERK1/2 and MLC. HEK293T cells were transfected with different amounts of HDAC6-flag for overnight and harvested for Western blot analysis as indicated.

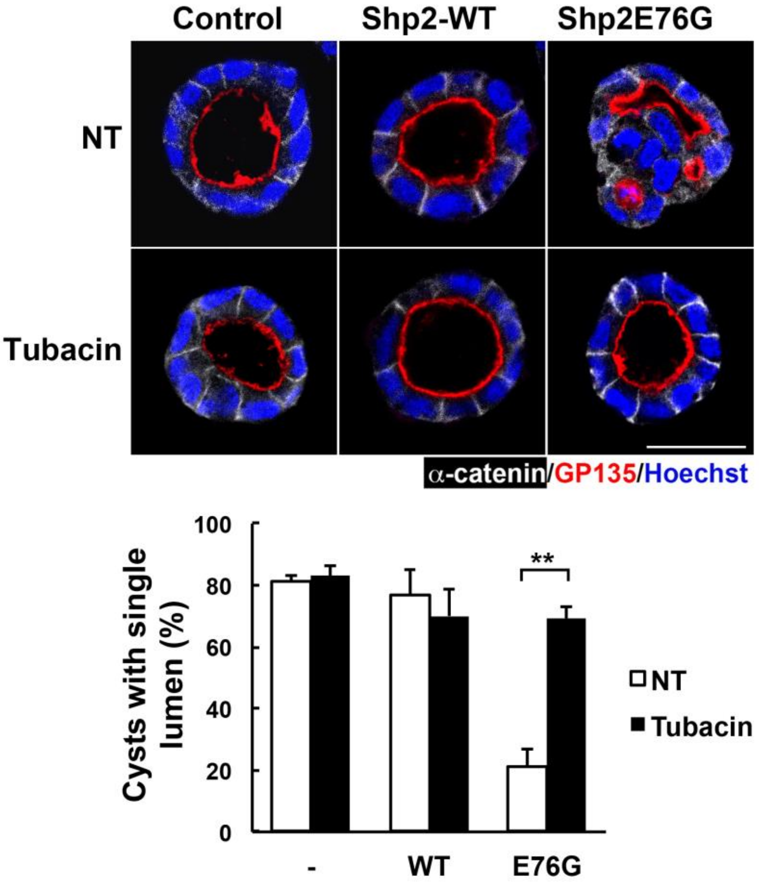

Supplementary Figure 5 Inhibition of HDAC6 restores normal lumen formation in Shp2-E76G cells. MDCK control cells and GFP-Shp2-WT and GFP-Shp2-E76G expressing cells were treated with or without 1 μM of tubacin treatment for 7 days. Cysts were fixed for IF staining with anti-α-catenin (white), anti-GP135 (red) antibodies and Hoechst (blue). The percentage of cysts with single lumen is shown at the bottom. Values are mean ± S.D from three independent experiments. **P<0.01 based on Student's t test. Scale bar, 20 μm.

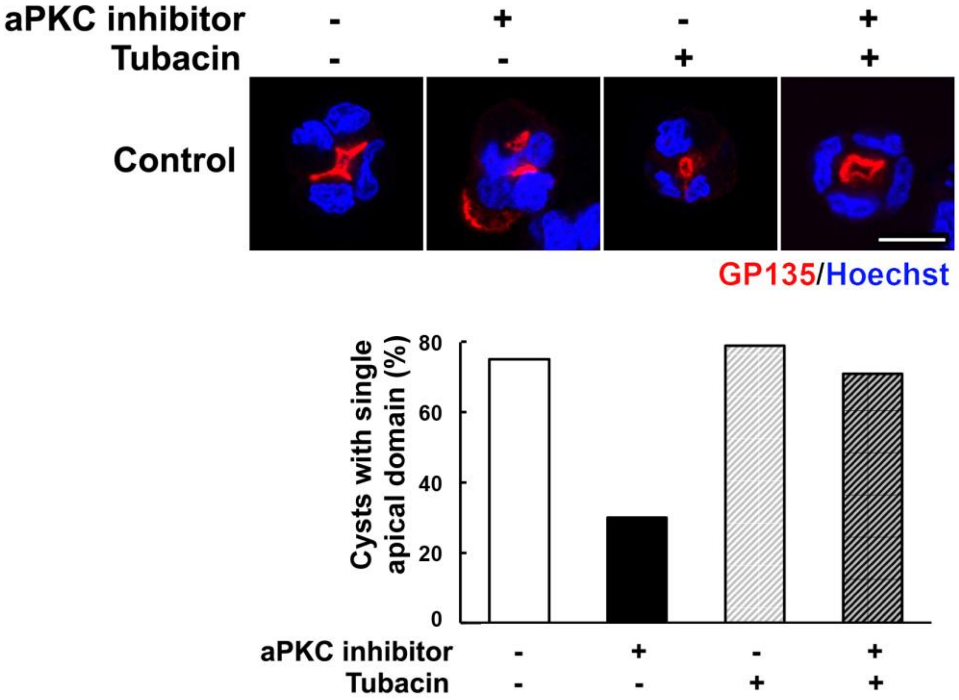

Supplementary Figure 6 Inhibition of HDAC6 overcomes aPKC blockage-induced multiple lumen. MDCK cells were cultured in Matrigel and co-treated with aPKC inhibitor (20 μM) with or without tubacin (1 μM) for 2 days. Cysts were fixed for IF staining with anti-GP135 antibody (red) and Hoechst (blue). The percentage of cysts with single apical domain were calculated from 200 cysts and shown at the bottom. Scale bar, 10 μm.

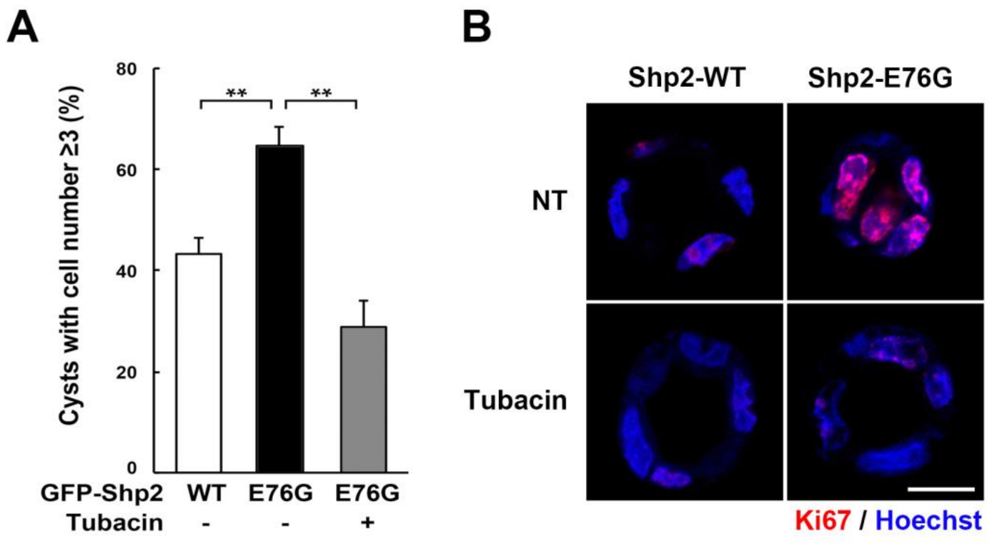

Supplementary Figure 7 Inhibition of HDAC6 restores oncogenic Shp2-induced abnormal growth. **(A)** MDCK cells embedded in Matrigel were treated with or without Tubacin (1 μM) for 36-48 h. Cysts were fixed and stained with Hoechst. The percentage of cysts with more than three cells were counted from 200 cysts and expressed in percentages (n=3). ** P<0.01 based on Student's t test. **(B)** MDCK cells were cultured in Matrigel and treated with or without tubacin (1 μM) for 2 days. Cysts were harvested and fixed for IF staining with anti-Ki67 (red) and Hoechst (blue). Scale bar, 10 μm.

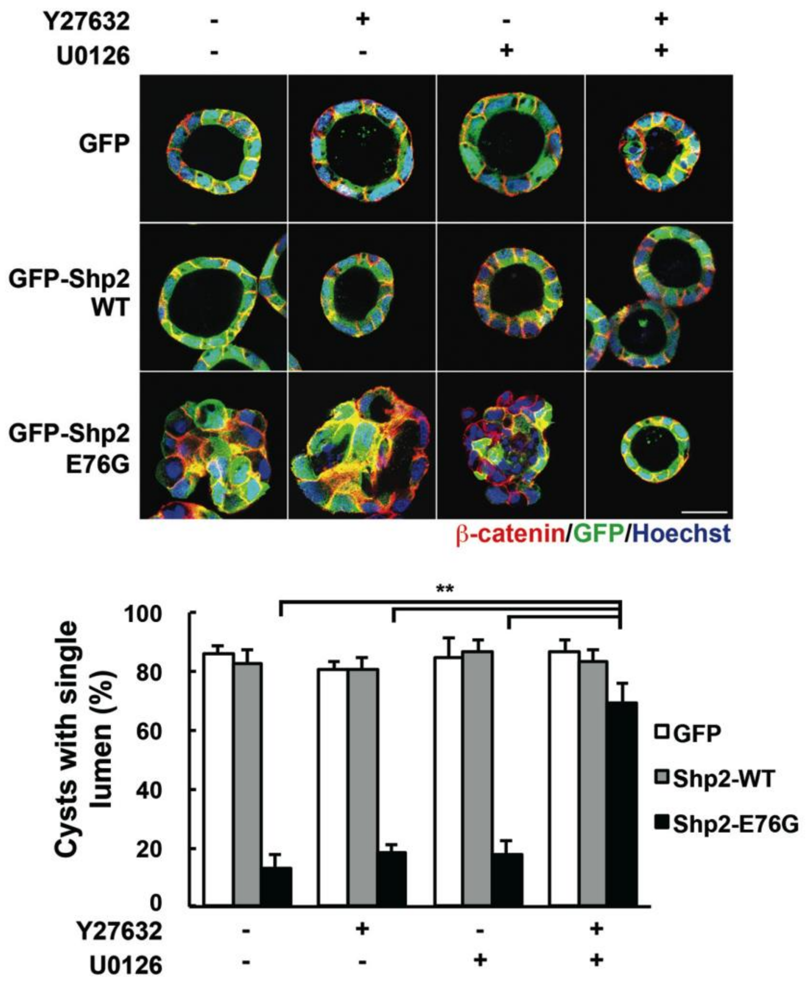

Supplementary Figure 8 Simultaneous inhibition of ROCK and ERK activity by Y27632 and U0126 restores normal lumen formation in E76G cysts. GFP-Shp2-WT and E76G expressing cells in Matrigel were treated with or without 10 μM of Y27632 or/and 10 μM of U0126 daily as indicated. Cysts were fixed for IF staining with antibodies as indicated. Scale bar, 20 μm. The percentage of cysts with single lumen is shown below (more than 500 cysts were counted in each experiment, n=3). ** P<0.01 based on Student's t test.

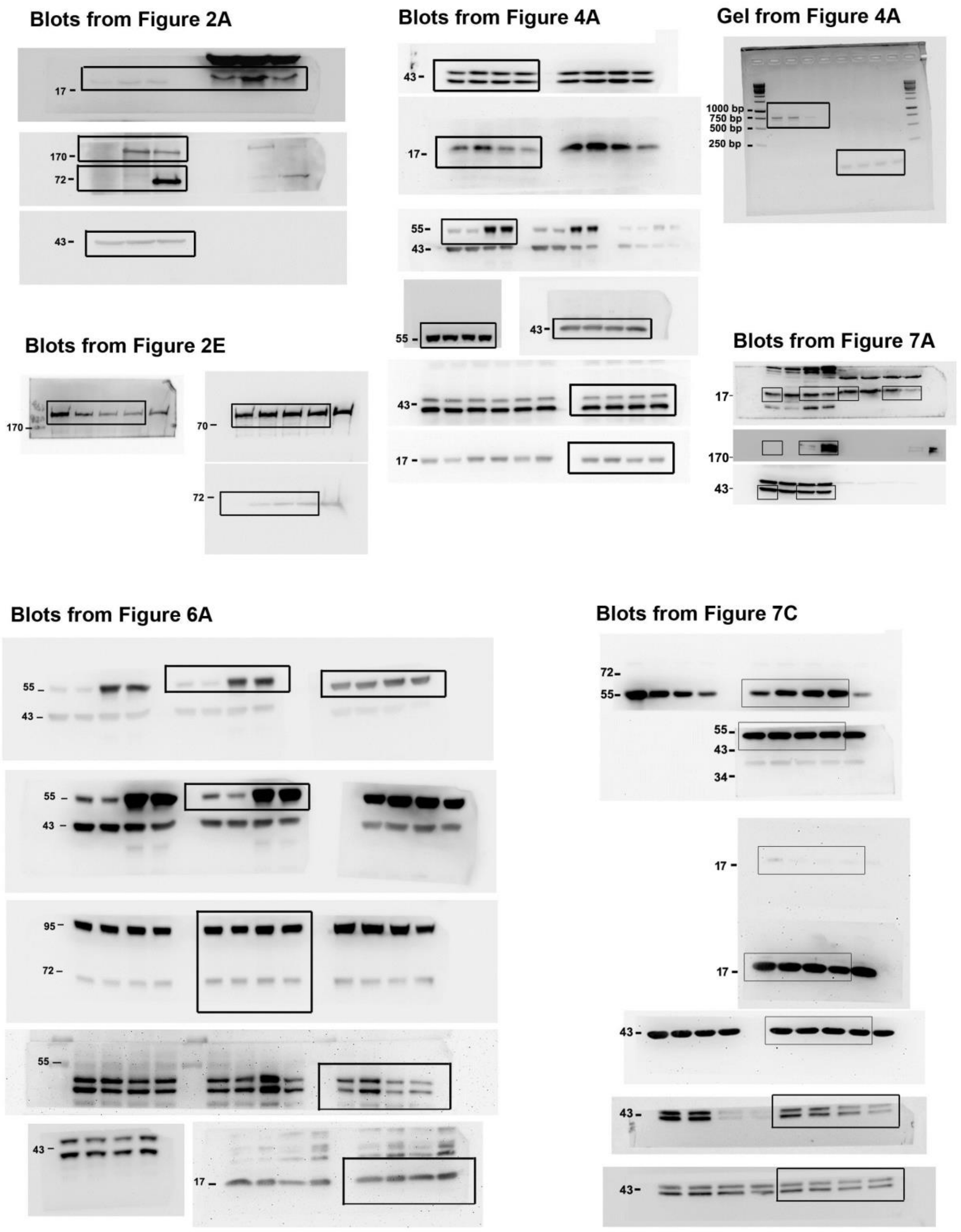

Supplementary Figure 9 Original uncropped Western blots.

Supplementary Methods

Mass analysis

Orbitrap Fusion Tribrid mass spectrometer (Thermo Scientific, San Jose, CA) was coupled with a Ultimate 3000 RSLCnano system (Thermo Fisher Scientific) with an HTC-PAL autosampler (CTC Analytics, Zwingen, Switzerland). Peptide mixtures were loaded onto a 100 μm × 150 mm fused-silica capillary column packed with C18 material (3 μm, Dr. Maisch GmbH, Amerbuch, Germany). The mobile phases consisted of (A) 0.1% formic acid and (B) 0.1% formic acid and acetonitrile. Peptides were separated through a gradient of up to 85% buffer B over 60 min at flow rate of 500 nL/min. The MS instrument was operated in the positive ion mode, with an electrospray through a heated ion transfer tube (275 °C), followed by a stacked ring ion guide (RF-lens) evacuated by a rotary vane pump to ∼2 Torr. Data were acquired in data-dependent acquisition mode with a top 15 method. Full-scan (m/z 320−2000) MS spectra were acquired at a target value of 4 × 105 and a resolution of 120,000, and the higher-collisional dissociation (HCD) tandem mass spectra (MS/MS) were recorded at a target value of 5 × 104 and with a resolution of 30,000 with a normalized collision energy (NCE) of 35%. The maximum MS1 and MS2 injection times both were 50 ms. The precursor ion masses of scanned ions were dynamically excluded (DE) from MS/MS analysis for 60 s. Ions with charge 1, and greater than 8 were excluded from triggering MS2 events. All raw data was analyzed using MaxQuant (version 1.5.0.30) and its integrated search engine Andromeda. The first search was carried out with 20 ppm, whereas the main search used 4.5 ppm for precursor ions. Mass tolerance of MS/MS spectra were set to 20 ppm to search against an in silicodigested UniProt reference proteome for Homo sapiens (Jun 2014, 20265 proteins) and 262 common mass spectrometry contaminants. The minimum

peptide length was set to 7. Label-free quantification was carried out using MaxLFQ with fast LFQ.
